# The Combination of Synoeca-MP Antimicrobial Peptide with IDR-1018 Stimulates Proliferation, Migration, and the Expression of Pro-Regenerative Genes in Both Human Skin Cell Cultures and 3D Skin Equivalents

**DOI:** 10.3390/biom13050804

**Published:** 2023-05-09

**Authors:** Thuany Alencar-Silva, Rubén D. Díaz-Martín, Alessandra Zonari, Daniel Foyt, Mylieneth Guiang, Robert Pogue, Felipe Saldanha-Araujo, Simoni Campos Dias, Octavio Luiz Franco, Juliana Lott Carvalho

**Affiliations:** 1 Programa de Pós-Graduação em Ciências Genômicas e Biotecnologia, Universidade Católica de Brasília, Brasília 71966-900, DF, Brazil; 2 OneSkin, Inc., San Francisco, CA 94107, USA; 3 Laboratório de Hematologia e Células-Tronco, Departamento e Farmácia, Universidade de Brasília, Brasília 70910-900, DF, Brazil; 4 Programa de Pós-Graduação em Patologia Molecular, Universidade de Brasília, Brasília 70910-900, DF, Brazil; 5 Programa de Pós-Graduação em Biologia Animal, Universidade de Brasília, Brasília 70910-900, DF, Brazil; 6 S-Inova Biotech, Pós-Graduação em Biotecnologia, Universidade Católica Dom Bosco, Campo Grande 79117-900, MS, Brazil; 7 Centro de Análises Proteômicas e Bioquímicas, Programa de Pós-Graduação em Ciências Genômicas e Biotecnologia, Universidade Católica de Brasília, Brasília 71966-900, DF, Brazil; 8 Laboratório Interdisciplinar de Biociências, Faculdade de Medicina, Campus Darcy Ribeiro, Universidade de Brasília, Brasília 70910-900, DF, Brazil

**Keywords:** Synoeca-MP, IDR-1018 peptide, antibacterial activity, wound healing, 3D skin equivalents

## Abstract

In skin lesions, the development of microbial infection affects the healing process, increasing morbidity and mortality rates in patients with severe burns, diabetic foot, and other types of skin injuries. Synoeca-MP is an antimicrobial peptide (AMP) that exhibits activity against several bacteria of clinical importance, but its cytotoxicity can represent a problem for its positioning as an effective antimicrobial compound. In contrast, the immunomodulatory peptide IDR-1018 presents low toxicity and a wide regenerative potential due to its ability to reduce apoptotic mRNA expression and promote skin cell proliferation. In the present study, we used human skin cells and a 3D skin equivalent models to analyze the potential of the IDR-1018 peptide to attenuate the cytotoxicity of synoeca-MP, as well as the influence of synoeca-MP/IDR-1018 combination on cell proliferation, regenerative processes, and wound repair. We found that the addition of IDR-1018 significantly improved the biological properties of synoeca-MP on skin cells without modifying its antibacterial activity against *S. aureus*. Likewise, in both melanocytes and keratinocytes, the treatment with synoeca-MP/IDR-1018 combination induces cell proliferation and migration, while in a 3D human skin equivalent model, it can accelerate wound reepithelization. Furthermore, treatment with this peptide combination generates an up-regulation in the expression of pro-regenerative genes in both monolayer cell cultures and in 3D skin equivalents. This data suggests that the synoeca-MP/IDR-1018 combination possesses a good profile of antimicrobial and pro-regenerative activity, opening the door to the development of new strategies for the treatment of skin lesions.

## 1. Introduction

Although the skin plays a vital role in protecting the body, its repair capacity is limited, and in situations of injury, the structure and function of this organ are compromised, which can result in the accumulation of degraded proteins, immune response dysfunction, and tissue loss [1]. This, added to other factors such as the high bacterial load on the skin and bacterial translocation, can promote the development of infectious complications, which consume additional resources, and are associated with significant morbidity and mortality in patients with severe burns, diabetic foot ulcers, and other skin injuries [2,3].

Synoeca-MP is an antimicrobial peptide (AMP) belonging to the mastoparan class, which exhibits excellent antimicrobial properties against several bacteria with clinical importance, such as methicillin-resistant *Staphylococcus aureus* (MRSA), vancomycin-resistant *Enterococcus faecalis*, *Pseudomonas aeruginosa*, and *Klebsiella pneumoniae* with relatively low minimum inhibitory concentrations (MIC; MIC = 3.5, 27.3, 8.3, and 10.1 μg/mL respectively) [4]. Despite its antibacterial properties, it has been observed that in the macrophage cell line RAW 264, synoeca-MP can induce a reduction of up to 60% in the cell viability at concentrations greater than 8 μg/mL, which limits its use in combating bacterial infections in vivo [5,6].

In contrast, the innate defense regulator peptide-1018 (IDR-1018), a synthetic peptide derivative from the host-defense peptide bactenecin, not only presents a moderate antibacterial activity but also has the ability to modulate the differentiation and activation of macrophages and neutrophils, thereby regulating their production of chemokines and cytokines [7,8,9]. Thanks to its immunomodulatory capability, IDR-1018 can promote wound healing via the modulation of tissue inflammation. Furthermore, this peptide has the capability to induce cell proliferation, migration, and reparative gene expression in a human skin equivalent 3D model, which suggests its potential for the treatment of skin wounds [8,10,11].

In the present study, using both a 2D culture system of the main cell types of the human skin (fibroblasts, keratinocytes, and melanocytes) and a 3D model of skin equivalents, we analyzed the potential of IDR-1018 peptide to attenuate synoeca-MP cytotoxicity without affecting the antibacterial activity of this AMP. Likewise, we evaluated the influence of the synoeca-MP/IDR-1018 combination on cell proliferation, migration, and the expression of regenerative-related genes.

Our results indicate that the biological effects of synoeca-MP on skin cells can be optimized by its combination with the IDR-1018 peptide. Likewise, we observed that the addition of IDR-1018 does not modify the antibacterial activity of synoeca-MP against *S. aureus*, the most common pathogen involved in skin infections. In addition, we observed that the treatment with synoeca-MP/IDR-1018 promotes cell proliferation and migration of human skin cells, and in the 3D human skin equivalent model, this peptide combination can facilitate a faster reepithelization, as well as a thicker epidermis development. Furthermore, the synoeca-MP/IDR-1018 treatment can induce changes in the expression patterns of the *FGF2*, *VEGFA*, *COL1A1*, *TGFB1*, and *TGFB3* genes that are related to the regenerative process.

These results suggest that IDR-1018 peptide protects human skin cells and 3D skin equivalents model against synoeca-MP cytotoxicity via stimulation of proliferation and induction of pro-regenerative genes without compromising the antimicrobial properties of synoeca-MP, constituting an exciting alternative for further studies in the skin regeneration.

## 2. Materials and Methods

### 2.1. Cell Lines and Maintenance

Human primary fibroblasts (Fibs) and keratinocytes (Kcs) were kindly provided by CellSeq Solutions, (Belo Horizonte, Brazil). The melanoma cell line HTB-65-ATCC was used as melanocyte cell type (MeWo). For the 3D human skin equivalent production, Fibs and Kcs were purchased from MatTek Corporation (Ashland, MA, USA). Fibs and MeWo were cultured in DMEM (Dulbecco’s Modified Eagle’s Medium, Gibco, Waltham, MA, USA) supplemented with 10% v.v. fetal bovine serum (FBS; Gibco, Waltham, MA, USA), and 1000 U/mL of penicillin/streptomycin solution (Invitrogen, Grand Island, NY, USA). Kcs were cultured in Keratinocyte Serum Free Media (KSFM, Gibco, Waltham, MA, USA). Cells were kept at 5% CO_2_, 37 °C, and 95% humidity.

### 2.2. Peptide Acquisition and Characterization

Synoeca-MP (INWLKLGKKIIASL) and IDR-1018 (VRLIVAVRIWRR) were purchased from Peptide 2.0 Inc. (Chantilly, VA, USA), and showed the expected molecular mass and >95% purity, as verified using matrix-assisted laser desorption Ionization Mass Spectrometry (MALDI-TOF Ultraflex III-Bruker Daltonics, Billerica, MA, USA). Briefly, peptides were resuspended in ultrapure water and analyzed in a matrix of α-cyano-4-hydroxycinnamic acid at the ratio of 1:3 (v.v.). The samples were then crystallized at room temperature (Anchorchip Var-384) for mass determination. Peptide Calibration Standard II was used for mass spectrometry to obtain the monoisotopic mass. Peptides were maintained in aliquots stored at −20 °C until use in order to avoid successive freezing and thawing.

### 2.3. Peptide Treatment

Synoeca-MP cytotoxicity was evaluated in human primary fibroblasts (Fibs) that were exposed for 24 h at 20, 40, 80, and 160 μg/mL of this peptide. The treatment of Fibs, Kcs, and MeWo with Synoeca-MP alone and in combination with IDR-1018 was performed for 48 h at 16 μg/mL for synoeca-MP and 64 μg/mL for IDR-1018. The experimental design included a positive control maintained in DMEM supplemented with 10% FBS and a negative control without FBS. For Kcs, this group was not included because KSFM media already lack FBS. The peptide treatments were performed on basal media lacking FBS, and all assays were performed in biologically independent triplicates.

### 2.4. Cell Viability and Proliferation

The viability and growth rate of Fibs, Kcs and MeWo were determined in 96-well plates by measuring 3-[4,5-dimethylthiazol-2-yl]-2,5-diphenyltetrazolium bromide metabolization using an MTT assay kit (Sigma Chemicals Co., St. Louis, MS, USA). Briefly, at 1, 4, and 7 days of treatment, 5 mg/mL of MTT was added to each well, and the plates were incubated for 4 h, protected from light. Formazan crystals were dissolved using DMSO for 15 min, and the optical density was determined in a microplate reader (Bio-Tec PowerWave, HT, USA) at 595 nm (and reference wavelength 650 nm).

### 2.5. Cell Migration Assay

Cell migration capacity was assessed by the wound scratch assay, according to Liang et al. (2007) [12]. In breve, human primary Fibs, Kcs and MeWo, were plated in 6-well plates and cultured until confluence. Then, scratches were made in the cell monolayers with 200 μL tips. Cell debris was removed by PBS washing, and then fresh cell culture media supplemented with peptide treatments were added to the wells. Cultures were incubated at 37 °C and documented at 0, 24, 48, and 72 h using a Zeiss Primo Vert microscope equipped with a digital camera (Carl Zeiss, Heidelberg, Germany). The number of cells that invaded the initially scratched area was counted using the ImageJ software (National Institutes of Health, Bethesda, MD, USA). Data were normalized to the positive migration control at 48 h (100%). Samples were analyzed as triplicates.

### 2.6. Gene Expression Analysis 

For qRT-PCR analysis, primers were designed and validated for efficiency, as previously described [11]. Different genes were assessed for 2D and 3D cultures. Genes assessed for cell monolayers were *CXCR4*, *CXCR7*, Elastin (*ELN*), *FGF2* (or basic *bFGF*), *MKI67* (or *Ki67*), *MMP1*, and *VEGFA* (or VEGF). In 3D skin equivalents, *MMP1*, *TGFB1*, *TGF**b**3*, *FGF2*, *EPN3* (*Epsin*), *PRDM1* (*Blimp1*), *COL1A1*, *CXCL8* (*IL-8*), and *VEGF* were assessed. *GAPDH* was assessed as a ubiquitous control. Total RNA was isolated using TRIzol^TM^ Reagent (Thermofisher, Waltham, MA, USA), following the manufacturer’s instructions. Total RNA amount and quality were determined using a NanoDrop 1000 spectrophotometer (NanoDrop, Wilmington, DE, USA) and Bioanalyzer (Agilent Genomics, Santa Clara, CA, USA). RNA samples were reverse-transcribed using the High-Capacity cDNA Reverse Transcription Kit (Thermofisher, Waltham, MA, USA), following the manufacturer’s instructions. qRT-PCR was performed in technical duplicates. Reactions were prepared with standardized reagents for real-time PCR (SYBR^TM^ Green Master Mix, Thermofisher, Waltham, MA, USA) added from primer sets specific to each gene or using Taqman probes (TaqMan™ Universal PCR Master Mix, Thermofisher, Waltham, MA, USA). Amplification reactions were performed using the StepOne Plus equipment (Applied Biosystems, Waltham, MA, USA). Ct values were determined using StepOne Software v2.3. The individual results expressed in Ct values were transferred to spreadsheets for 2^−ΔΔCT^ analysis. Primer sequence and Taqman probe information are shown in Table S1.

### 2.7. Human 3D Skin Equivalent Wound Models

Full-thickness skins were produced according to Zonari et al. (2020) [13]. Briefly, dermal models were built using rat tail collagen type I (Corning Inc. Corning, NY, USA), 10X HAM-F12 (Gibco Inc. Waltham, MA, USA), and 10X reconstitution buffer embedded with 1.5 × 10^4^ Fibs. After polymerization and the addition of DMEM 10% FBS, the collagen lattices were equilibrated for 1–2 h and seeded with 1.5 × 10^5^ Kcs on top. After 24 h submerged culture, the human skin equivalents were transferred to an air-liquid interface and maintained for 7 additional days to promote epidermal differentiation and stratification. The wounds were made by punching the full-thickness skin equivalents with a 2.5 mm circular biopsy punch and placing the wound skin equivalents on top of a new dermal compartment produced as described. The wound area was treated with 20 μL of either: fibrin gel (2.5 μL of fibrinogen at 40 μg/mL, 7.5 μL of water and 10 μL of thrombin at 25 U/mL), fibrin gel added with 16 μg/mL of synoeca-MP and 64 μg/mL of IDR-1018. Peptide treatment was also performed by adding the same concentration of the peptides in the medium. Treatment was performed immediately after lesioned model production and 3 days later and wound healing was observed for 3 and 5 days. At those time points, 3 equivalents per group were fixed in 10% v.v. formalin, and paraffin-embedded. Representative sections were stained with a hematoxylin and eosin stain kit (Vector Laboratories, Newark, CA, USA) following routine protocol. Another 3 skins per group were used for qRT-PCR analysis.

### 2.8. Antimicrobial Assays for Inhibitory and Bactericidal Concentrations against S. aureus

Minimal inhibitory concentration (MIC) of synoeca-MP and IDR-1018 and the combination of both were determined by microdilution broth technique for aerobic bacteria [14]. To analyze bacterial growth, fresh colonies of *S. aureus* (25,923 strain) were inoculated in Luria-Bertani (LB) medium (Kasvi, São José dos Pinhais, Brazil) and incubated for 16 h at 220 rpm and 37 °C. Bacterial growth was monitored until the absorbance at 600 nm reached 0.7 OD (4 × 10^8^ CFU.mL^−1^) corresponding to the log phase. The bacterial suspension was diluted to a final concentration of 5 × 10^4^ UFC/mL in each well (96-well plates). For antimicrobial assays, synoeca-MP and IDR-1018 were tested in a range from 1 to 128 μg/mL. All antimicrobials and bacterial suspension were seeded together, and ampicillin (20 μg/mL) was used as a positive control. The assays were performed in three independent experiments, and the absorbance was monitored in a microplate reader (BioTek Power Wave HT, Winooski, VT, USA). After incubation (18 to 24 h), a suspension of 10 μL of wells with inhibitory concentrations (MIC, no growth determined by absorbance) was inoculated in LB agar and for confirmation of bactericidal (MBC, no growth after incubation) or bacteriostatic activity (MIC, some growth after incubation).

### 2.9. Drug Combination-Adapted Checker-Board Assay

Synoeca-MP and IDR-1018 were tested against *S. aureus* (25,923 strain), and combinations in several concentrations were assessed using the checkerboard method [15]. The susceptibility tests were performed using the microdilution method, as explained above. Peptides and drug combinations were analyzed against bacterial suspension of *S. aureus* (2 × 10^6^ CFU/mL) in a 96-well plate. The chosen concentrations for testing were 16 μg/mL for synoeca-MP and 2–64 μg/mL for IDR-1018. Bacterial growth was observed in a microplate reader at 600 nm after incubation for 24  h at 37 °C. The fractional inhibitory concentration index (FICI) was calculated.

### 2.10. Statistical Analysis

All experiments were performed in three or more biologically independent experiments, in addition to technical replicates, detailed in each assay. Data were analyzed using analysis of variance and independent sample t-test with the software GraphPad Prism^®^ Software, version 7.02 (San Diego, CA, USA, 2017). A *p*-value of less than 0.05 was considered significant.

## 3. Results

### 3.1. IDR-1018 Did Not Compromise the Antibacterial Effect of Synoeca-MP 

To analyze the possible effect of IDR-1018 on the well-characterized antimicrobial activity of synoeca-MP, *S. aureus* 25,923 was exposed to synoeca-MP in the presence of IDR-1018. The chosen concentration of IDR-1018 was 64 μg/mL since, at this concentration, this peptide has immunomodulatory and regenerative effects without cytotoxic effects in human cells [11,16]. Our results show that synoeca-MP exhibits microbicidal activity against the tested strain of *S. aureus* at concentrations equal to or greater than 16 μg/mL (Table 1). In contrast, in our hands, IDR-1018 did not show an antimicrobial effect against *S. aureus* at any of the concentrations tested (1 to 128 μg/mL). The MBC assay confirmed the MIC of synoeca-MP against *S. aureus* at 16 μg/mL (Table 1). The checkerboard test to analyze the interaction between these two molecules reveals that there is no antagonism when synoeca-MP (16 μg/mL) is combined with IDR-1018 in a range of 16 to 64 μg/mL (Table 2). Suggesting that IDR-1018 did not compromise the bactericidal effect of synoeca-MP that maintains its MIC at 16 μg/mL. Interestingly, three potential synergistic interaction points were found between synoeca-MP at 16 μg/mL and IDR-1018 at 2, 4, and 8 μg/mL, but this result is not conclusive since we observed that IDR-1018 had no MIC against *S. aureus* at any of the concentrations tested. Based on these results, for further experiments, we still kept the concentrations of 16 μg/mL for synoeca-MP and 64 μg/mL for IDR-1018.

### 3.2. Cytotoxic Potential of Synoeca-MP on Skin Cells

**Figure 1:**
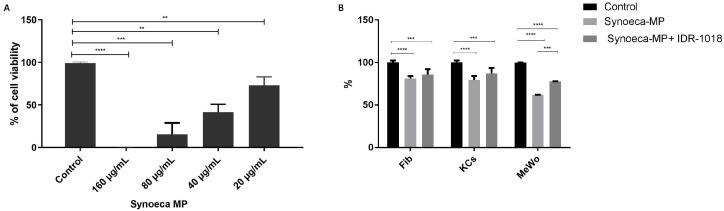
**IDR-1018 reduces synoeca-MP cytotoxicity on skin cells**. The cytotoxic potential of synoeca-MP on skin cells was tested in fibroblast (Fibs) at concentrations of 20, 40, 80, and 160 μg/mL for 24 h (**A**). The effect of IDR-1018 on the cytotoxic profile of synoeca-MP was tested in Fibs, keratinocytes (Kcs), and melanocytes (MeWo). Cells were incubated with synoeca-MP alone (16 μg/mL) or in combination with IDR-1018 (64 μg/mL) for 24 h (**B**). The mean ± SD of three independent experiments is plotted. Comparisons among groups were conducted with one-way ANOVA and Tukey’s post hoc tests. Asterisks indicate significant differences (** *p* < 0.01; *** *p* < 0.001, **** *p* < 0.0001) with regard to the control.

To analyze the potential cytotoxic effect of synoeca-MP against skin cells, Fibs were treated with synoeca-MP at 20, 40, 80, and 160 μg/mL (Figure 1A). At 24 h of peptide treatment, we observed a significant reduction in cell viability at concentrations of 40, 80, and 160 μg/mL (56.2% ± 6.1 *p* < 0.05; 67.7% ± 5.96 *p* < 0.05; and 100% *p* < 0.05 respectively). In contrast, the Fibs exposed to synoeca-MP at 20 μg/mL only present a reduction of cell viability of 22.1% ± 5.72 (*p* < 0.05) in comparison with the unexposed control group.

### 3.3. IDR-1018 Peptide Reduces Synoeca-MP Cytotoxicity

In order to determine the effect of IDR-1018 on the cytotoxic profile of synoeca-MP, different human skin cell types (Fibs, Kcs, and MeWo) were incubated with synoeca-MP alone (16 μg/mL) or in combination with IDR-1018 (64 μg/mL) for 24 h (Figure 1B). Synoeca-MP exposure induced a significant reduction in cell viability of the three cell types tested in relation to the not-exposed control (Fibs = 77.4% ± 6.1; Kcs = 84.41% ± 5.52; MeWo = 62.02% ± 1.2 *p* < 0.05). In contrast, the presence of IDR-1018 generates a reduction in cytotoxicity, reflected in an increase of cell viability in the three cell types tested (Fibs = 87.87% ± 5.96; Kcs = 93.28% ± 5.72; MeWo = 73.63% ± 2.45 *p* < 0.05). Interestingly, in MeWo, the addition of IDR-1018 generated a significant increase in cell viability compared to the group exposed to synoeca-MP alone (from; 62.02% ± 1.2 vs. 73.63% ± 2.45, *p* < 0.05).

### 3.4. Effects of Synoeca-MP/IDR-1018 in Cell Proliferation and Migration

**Figure 2:**
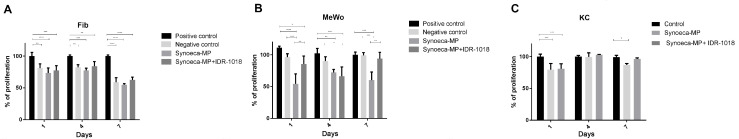
**Effects of synoeca-MP/IDR-1018 on cell proliferation.** Cells (**A**): fibroblast (Fibs), (**B**): keratinocytes (Kcs), and (**C**): melanocytes (MeWo) were incubated with synoeca-MP/IDR-1018 combination (16 and 64 μg/mL respectively), and the cell proliferation was determined at 1, 4, and 7 days by MTT assay. The mean ± SD of three independent experiments is plotted. Comparisons among groups were conducted with a one-way ANOVA and Tukey’s post hoc test. Asterisks indicate significant differences (* *p* < 0.05; ** *p* < 0.01; *** *p* < 0.001, **** *p* < 0.0001) with regard to the control.

To analyze the effect of the synoeca-MP/IDR-1018 combination on the proliferative potential of skin cells, we determined cell proliferation at 1, 4, and 7 days in both Fibs, Kcs and MeWo incubated with this peptide combination (Figure 2). Synoeca-MP alone was not effective in supporting cell proliferation in any of the cell types tested (Fibs 56.62% ± 1.81; Kcs 89.36% ± 2.65; MeWo 91.15% ± 2.23, *p* < 0.05, normalized to positive proliferation controls at day 7). In Fibs, synoeca-MP/IDR-1018 treatment did not result in any increase in cell proliferation, compared to the group treated with synoeca-MP alone (56.62% ± 1.81 vs. 66.93% ± 4.42, normalized to the positive proliferation control; Figure 2A). In contrast, the treatment of Kcs and MeWo with the combination of peptides (Figure 2B,C) allowed cell proliferation to be maintained at levels similar to those observed in the control group at 7 days of treatment (Kcs 97.82% and 93.91% ± 6.5), respectively, normalized to positive proliferation controls).

**Figure 3:**
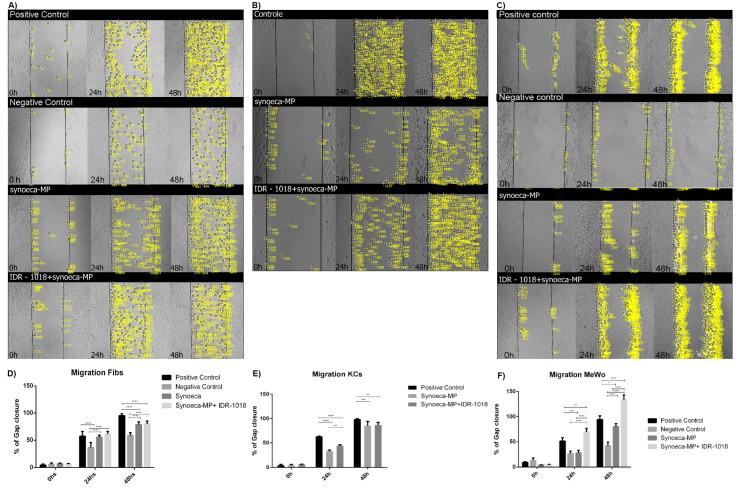
**Effects of synoeca-MP/IDR-1018 on migratory capability.** The effect of treatment with synoeca-MP/IDR-1018 combination on cell migration was tested in fibroblast (Fibs: (**A**,**D**)), keratinocytes (Kcs: (**B**,**E**)), and melanocytes (MeWo: (**C**,**F**)). Yellow dots represent the cells that are migrating to close the gap. Positive control was incubated in a basal medium supplemented with FBS, while negative control was incubated in a basal medium without FSB. The mean ± SD of the percentage of cells that migrated in three independent experiments at 0, 24, and 48 h is represented. Comparisons among groups were conducted with one-way ANOVA and Tukey’s post hoc tests. Asterisks indicate significant differences (* *p* < 0.05; ** *p* < 0.01; *** *p* < 0.001, **** *p* < 0.0001) with regard to the control.

At 24 h the treatment of Fibs with synoeca-MP/IDR-1018 (Figure 3A,D) can induce a similar migratory capability than the observed in the positive control (56.88% ± 4.3 control vs. 60.53% ± 2.44 synoeca-MP/IDR-1018 *p* < 0.05).

In contrast, at 48 h, this treatment induces a lower migratory percentage than that observed in the positive control (100% control vs. 80.6% ± 2.55 synoeca-MP/IDR-1018 *p* ≤ 0.0001), suggesting that the synoeca-MP/IDR-1018 combination can only partially support cell migration in this cell type. On the other hand, the treatment of Kcs with synoeca-MP/IDR-1018 at both 24 and 48 h can generate a higher migratory capability than that observed in the group treated with synoeca-MP alone (46.13% ± 3.12 at 24 h and 92.58% ± 7.02 at 48 h, *p* < 0.05; Figure 3B,E).

Interestingly at both 24 and 48 h, the treatment of MeWo with synoeca-MP/IDR-1018 increase the migratory capabilities in comparison to the positive control (Figure 3C,F), suggesting that this peptide combination can induce cell migration (51.45% ± 1.4 control vs. 69.45% ± 7.39 synoeca-MP/IDR-1018 at 24 h, *p* < 0.05, and 94.27 ± 6.2 control vs. 133.42% ± 9.82 synoeca-MP/IDR-1018 at 48 h *p* < 0.05).

### 3.5. Synoeca-MP/IDR-1018 Can Modify the Expression of Pro-Regenerative Genes

**Figure 4:**
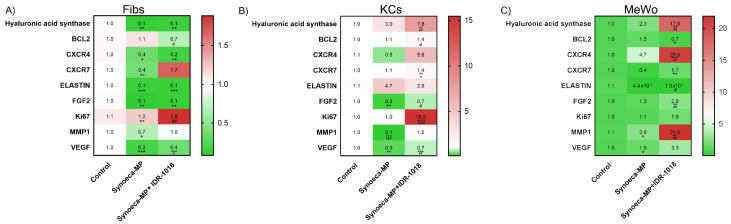
**Synoeca-MP/IDR-1018 can modify the expression of pro-regenerative genes.** Relative mRNA expression profile of Fibroblasts (Fibs: (**A**)), keratinocytes (Kcs: (**B**)), and melanocytes (MeWo: (**C**)) treated with synoeca-MP/IDR-1018 was analyzed at 48 h of treatment. Expression levels correspond to the color bar on the right side of the heat map. The mRNA assessed corresponds to hyaluronic acid synthase 2 (*HAS2*), C-X-C chemokine receptor type 4 (*CXCR4*), C-X-C chemokine receptor type 7 (*CXCR7*), elastin, fibroblast growth factor 2 (*FGF2*), a marker of proliferation Ki-67 (*MKI67*), matrix metalloproteinase 1 (*MMP1*), vascular endothelial growth factor (VEGF). Glyceraldehyde 3-phosphate dehydrogenase (*GAPDH*) was used as a ubiquitous control. The analysis of the mean ± SD of three independent experiments is represented. Comparisons among groups were conducted with one-way ANOVA and Dunnet’s post hoc tests. Asterisks indicate significant differences (* *p* < 0.05; ** *p* < 0.01; *** *p* < 0.001; **** *p* < 0.0001) with regard to the control. Hashtags indicate significant differences (# *p* < 0.05; ## *p* < 0.01; ### *p* < 0.001; #### *p* < 0.0001) with regard to synoeca-MP group.

To analyze how synoeca-MP/IDR-1018 treatment affects the skin regeneration process, we analyzed by qRT-PCR the expression of several pro-regenerative genes (Figure 4). In MeWo (Figure 4C), the treatment with synoeca-MP/IDR-1018 generates an increase in the expression levels of *HAS2*, *MMP1*, *FGF2*, *CXCR4*, and *CXCR7* genes (17.9 ± 1.5, 21.9 ± 0.9; 2.6 ± 2.5; 20.9 ± 1.3; 1.7 ± 0.5 *p* < 0.01 respectively), which is accompanied by a slight increase in *VEGF* expression.

Likewise, in Kcs (Figure 4B), the treatment with this combination of peptides can generate a significant up-regulation in the expression level of *CXCR7*, *HAS2*, and *KI67* genes (1.4 ± 0.5; 7.8 ± 1.1; 15.5 ± 2.3, *p* < 0.01 or *p* < 0.001 respectively). In contrast, despite the fact that in Fibs (Figure 4A), the treatment with synoeca-MP/IDR-1018 generates the up-regulation of *KI67* (1.9 ± 0.3 *p* < 0.01), in this kind of cells, this treatment also induces the down-regulation of CXCR4, elastin, and HAS2 genes (0.2 ± 0.001; 0.1 ± 0.005; 0.1 ± 0.025; *p* < 0.01 and *p* < 0.001, respectively).

### 3.6. Synoeca-MP/IDR-1018 Promoted Skin Wound Healing in a 3D Skin Equivalent Model

**Figure 5:**
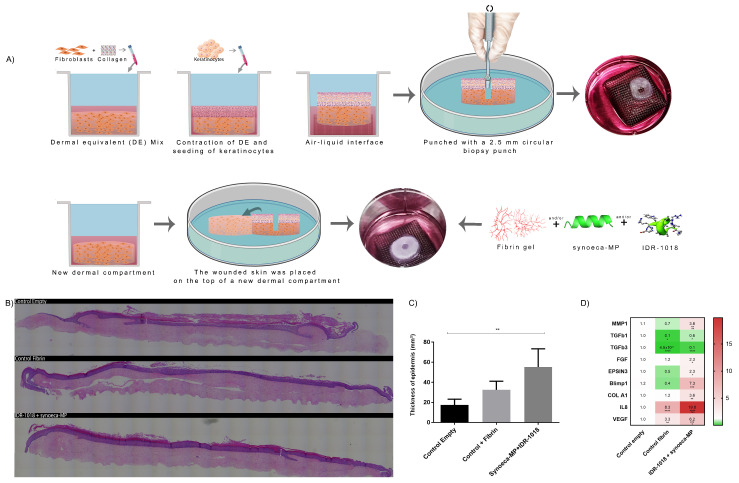
**Effects of synoeca-MP/IDR-1018 on reepithelization in a 3D skin equivalent model.** The 3D skin equivalents were wounded, and the effect of synoeca-MP/IDR-1018 treatment on re-epithelialization was analyzed. Summary of the experiment carried out in a 3D skin equivalent model (**A**) Lesion closure of the three-dimensional skin equivalents was assessed through histology (**B**). The epidermal thickness of the skin equivalents was quantified (**C**). Changes in the gene expression profile of the lesioned 3D skin equivalents were determined (**D**). Expression levels correspond to the color bar on the right side of the heat map. The genes assessed were: matrix metalloproteinase 1 (*MMP1*), tumor growth factor beta 1 (*TGFβ1*), tumor growth factor beta 3 (*TGFβ3*), fibroblast growth factor 2 (*FGF2*), Epsin 3, B lymphocyte-induced maturation protein 1 (*BLIMP1*), collagen type IA (*COL A1*), interleukin-8 (*IL-8*), vascular endothelial growth factor (*VEGF*). Glyceraldehyde 3-phosphate dehydrogenase (*GAPDH*) was used as a ubiquitous control. The analysis of the mean ± SD of three independent experiments is represented. Comparisons among groups were conducted with one-way ANOVA and Dunnet’s post hoc tests. Asterisks indicate significant differences (* *p* < 0.05; ** *p* < 0.01; *** *p* < 0.001, **** *p* < 0.0001) with regard to the control (empty).

To investigate the effect of the synoeca-MP/IDR-1018 combination in the wound healing process, we induced full-thickness lesions in a 3D human skin equivalent model by completely removing the epidermis and partially the dermis (Figure 5A,B). The treatment with fibrin plus synoeca-MP/IDR-1018 induced a better average epidermal thickness in comparison with both the control with fibrin and the control with culture medium alone (empty; 64 ± 11.3 mm; 36 ± 8.5 mm; 20 ± 5.69 mm respectively, *p* < 0.004 compared to the empty control), suggesting that synoeca-MP/IDR-1018 can induce a faster epithelization of the wound.

In the 3D human skin equivalent models, the gene expression analysis (Figure 5C) shows that the treatment with synoeca-MP/IDR-1018 can induce the down-regulation in the expression of *TGFb1* and *TGFb3* genes (0.6 ± 0.02 and 0.1 ± 0.05 respectively, *p* < 0.05), which is markedly lower than that observed in the group treated with topical fibrin (0.1 ± 0.003 *p* < 0.05, and 4.5 × 10^−^³ ± 0.00001 *p* < 0.001 respectively). Furthermore, the treatment with this peptide combination induced an up-regulation of *COL1A1*, *MMP1*, *EPSIN*, *IL8*, *FGF, VEGF*, and *BLIMP1* genes (3.6 ± 1.1; 3.8 ± 0.9; 2.3 ± 0.88; 19.8 ± 3.2, 2.3 ± 1.3; 6.2 ± 1.22, 7.3 ± 1.5, *p* < 0.05, or *p* < 0.0.1 or *p* < 0.001 respectively).

## 4. Discussion

Secondary bacterial infections in skin lesions are among the most common infections and may lead to serious local and systemic complications. These infections can be potentially life-threatening and may progress rapidly, hindering the process of skin regeneration. Therefore, searching for new therapeutic strategies that prevent the establishment of bacterial infections and promote skin regeneration is a key step to the proper management of this problem [17,18].

In this work, we used human skin cells and a 3D skin equivalent model to analyze the potential of the IDR-1018 peptide to attenuate the cytotoxicity of the antimicrobial peptide synoeca-MP, as well as the influence of synoeca-MP/IDR-1018 combination on cell proliferation, tissue repair, and in the expression of pro-regenerative genes.

It has been previously suggested that the peptide IDR-1018 does not affect the antibacterial activity of synoeca-MP [6]. In this work, we found that the immunoregulatory peptide IDR-1018 does not generate any antagonistic action that compromises the antimicrobial properties of synoeca-MP, and for the first time, we determined the antimicrobial effect of synoeca-MP against *S. aureus*, the highest pathogenic agent related to secondary infection of skin wounds [19].

Due to the decrease in the effectiveness of antibiotics by the arrival of resistant bacteria strains, it is urgent the search for new strategies for antibacterial treatment [20]. The relatively low MICs of synoeca-MP against resistant bacteria strains, such as methicillin-resistant *S. aureus* and vancomycin-resistant *E. faecalis*, open the possibility of positioning this AMP as a promising adjuvant to antibiotic therapies commonly used in the management of infections [4,5].

Despite the potential of synoeca-MP as an antimicrobial compound, the treatment of mammalian cells with concentrations close to the doses with antibacterial activity can generate a significant reduction in cell viability [5,6]. The analysis of the synoeca-MP cytotoxicity against skin cells showed moderate cytotoxicity at 16 and 20 μg/mL, suggesting that a strategy is necessary to reduce the cytotoxicity of this AMP without compromising its antibacterial activity.

Peptides with anti-inflammatory properties can protect against the effect of cytotoxic compounds. Fish scale collagen peptides (FSCP) protect human keratinocytes against CoCl_2_-induced cytotoxicity and TNF-α-induced inflammatory responses by the increase of cell viability and the reduction of oxidative stress through mechanisms mediated by the down-regulation of key proinflammatory cytokines [21]. Likewise, anti-inflammatory proline-rich peptides from the jararaca snake venom (Bj-PROs) protect neuroblastoma cell line SH-SY5Y against cytotoxicity induced by oxidative stress of H_2_O_2_, increasing cell viability by a mechanism related to decreasing ROS production [22]. We observed that treatment with the antiinflammatory peptide IDR-1018 attenuates the cytotoxic effects of synoeca-MP, both in Fibs, Kcs and MeWo, increasing cell viability in the group treated with the combination of peptides compared to the group treated with synoeca-MP alone.

IDR-1018 presents immunoregulatory properties related to the increase in intracellular calcium mobilization and the induction of cell chemotaxis by the activation of G-protein, phospholipase C, ERK, p38, JNK, and NF-κB signaling [7,23]. Interestingly, the toxicity mechanism proposed for mastoparans such as synoeca-MP is based on the destabilization of the eukaryotic cell membrane by the formation of pores, which can induce a change in the distribution and mobilization of Ca^2+^ to and from the reticulum, which affects the activation of several signaling pathways [24]. This suggests that the reduction in the cytotoxic effects of synoeca-MP induced by IDR-1018 may be related to the regulation of calcium mobilization, which promotes cell proliferation.

In addition to the immunoregulatory activity of IDR-1018, this peptide can induce cell migration and the up-regulation of proliferative-related genes. This peptide also promotes the production of specific pro and anti-inflammatory mediators associated with wound healing [10,11]. Our results showed that synoeca-MP/IDR-1018 treatment generates a higher migratory capability in both Kcs and MeWo, and in the particular case of melanocytes, the treatment with this combination of the peptides results in a significant increase in migratory capabilities that even exceeds that of the positive control.

Interestingly, it has been reported that IDR-1018 can significantly accelerate wound healing in porcine and murine models through a mechanism related to the reduction of inflammation [10]. Furthermore, IDR-1018 was shown to directly stimulate human keratinocyte proliferation and faster re-epithelialization of lesioned human 3D skin equivalents [11]. Our analysis showed that synoeca-MP/IDR-1018 combination has the potential to promote cell proliferation and induce a faster reepithelization of the wound in the 3D skin equivalent model.

It has been reported that the association of synoeca-MP with otter compounds such as levofloxacin may induce changes in the expression pattern of pro and anti-inflammatory mediators, showing that synoeca-MP may have immunoregulatory properties [6]. Therefore, it is possible that the synoeca-MP/IDR-1018 treatment allows keeping the pro-regenerative effects of IDR-1018 while maintaining the anti-inflammatory effect of both peptides. It has been reported that several anti-inflammatory peptides with regenerative properties, such as the tripeptide GHK (glycyl-L-histidyl-L-lysine), possess antioxidant effects, which supports cellular stemness and the secretion of trophic factors related to the regenerative activity of this kind of peptides [25]. Interestingly, it has been shown that the up-regulation in the expression of *FGF* and *IL-8*, which is induced by treatment with synoeca-MP/IDR-1018, may be related to an induction of the modulation of Ca2+ homeostasis and may attenuate the oxidative stress [26,27]. In this sense, several of the peptides present in the venom of various wasp species can effectively reduce oxidative stress and affect LPS-induced nitric oxide (NO) production, which is related to the reduction of reactive oxygen species (ROS) that may be involved in the cascade of events that lead to the induction of apoptosis [28], suggesting that one of the possible mechanism by which these peptides exert their anti-inflammatory effects is through the reduction of oxidative stress.

The expression analysis of several pro-regenerative genes reveals that the treatment with synoeca-MP/IDR-1018 promoted a balance in the regulation of both TGFβ1 and TGFβ3. Interestingly, it has been observed that other pro-regenerative peptides such as OA-GL12 or Tylotoin, which promote wound healing and acceleration of the scratch-healing of keratinocytes and human fibroblasts, have the ability to induce the production of TGF-β family members [29,30], a group of growth factors involved in a number of processes in wound healing such as inflammation, stimulating angiogenesis, fibroblast proliferation, collagen synthesis and deposition and remodeling of the new extracellular matrix [31,32]. Likewise, it is possible that the increase in the expression of TGFβ1 and TGFβ3 generated after treatment with the synoeca-MP/IDR-1018 combination is related to the increase in dynamic ECM regulation represented by the induction of *COL1A1*, *HAS2*, and *MMP1* expression. Similarly, other antimicrobial peptides which have pro-regenerative properties, such as the Human neutrophil peptide-1 (HNP-1), can alter the expression and function of ECM components, including collagen types I and III, and the metalloproteinase 1 (MMP-1), suggesting that this type of bioactive peptides may promote wound repair by enhancing extracellular matrix deposition and by controlling its degradation [33,34].

Although the regenerative potential of IDR-1018 to induce skin cell proliferation has been previously observed in different study models [9,10,11], the ability of the combination of IDR-1018 with synoeca-MP to induce the up-regulation of *Ki67*, *Epsin*, *IL-8*, and, *FGF*, suggests that this treatment is capable of maintaining the regenerative potential of IDR-1018, possibly through the regulation of the process of inflammation, which can not only be crucial to the control of invading microorganisms, but also to the production of various growth factors required to guide the process of re-epithelialization, fibroblast reconstruction, and ECM remodeling [11,35].

CXCR4 and CXCR7 are factors related to cell adhesion, cell migration, revascularization and chemotaxis, which are key events in tissue repair [35]. The up-regulation of *CXCR4* and *CXCR7* induced by synoeca-MP/IDR-1018 suggests that the combination of these peptides can modify the CXCR4 signal axis activity, which can increase the expression of cell adhesion and migration molecules necessary for the correct regulation of wound healing [36]. Likewise, CXCR4 and CXCR7 are involved in cell proliferation, which is the basis of tissue regeneration. It has been observed that these factors play a major role in liver regeneration and re-epithelialization by promoting stem cell migration and proliferation [37,38], which suggests that the increase in the expression of *CXCR4*, *CXCR7* induced by synoeca-MP/IDR-1018 treatment, may be a key factor in the promotion of skin cell migration, and proliferation induced by this combination of peptides [39].

## 5. Conclusions

In a complex scenario such as skin regeneration, the enhancement of cell proliferation, the regulation of the inflammatory process, and the prevention of microbial infection are key factors for the successful management of patients with severe skin injuries. IDR-1018 peptide can reduce the cytotoxic potential of the antimicrobial peptide synoeca-MP without affecting its antibacterial properties against *S. aureus*, one of the major etiological agents of skin wound complications. Likewise, the combination of synoeca-MP plus IDR-1018 promotes the proliferation and migration of skin cells and the expression of pro-regenerative genes. In addition, this combination of peptides can induce faster epithelization in a 3D skin equivalent model. Therefore, these results open the door to the development of new strategies for the treatment of skin lesions.

## Figures and Tables

**Table 1:** Antimicrobial activity of synoeca-MP and IDR-1018 peptides against *S. aureus* 25923. Determination of MIC and MBC values was performed at concentrations from 1 to 128 μg/mL, and ampicillin (20 μg/mL) was used as a positive control.

Antimicrobial Agent	MIC (μg/mL)	MBC (μg/mL)
Synoeca-MP	16	16
IDR-1018	Nd	Nd

**Table 2:** Synergistic results of synoeca-MP plus IDR-1018.The antimicrobial activity of synoeca-MP plus IDR-1018 peptides against *S. aureus* 25923 was determined. The assay was performed at concentrations from 2 to 64 μg/mL for IDR-1018 and at a constant concentration of 16 μg/mL for synoeca-MP. Note: FICI ≤ 0.5: synergism; FICI > 0.5 and <1.0: additive; FICI > 1.0 and ≤4: indifferent.

Isolated MIC	Tested Concentrations (μg/mL)					
Synoeca-MP (16 μg/mL)	16	16	16	16	16	16
IDR-1018 (>128 μg/mL)	2	4	8	16	32	64
FICI	0.28	0.31	0.375	0.5	0.75	1.25

## Data Availability

No new data were produced or analyzed in this study. Data sharing is not applicable to this article.

## Supplementary Materials

The following supporting information can be downloaded at: https://www.mdpi.com/article/10.3390/biom13050804/s1, Table S1. Primers and probes.
