# Supplementary material for: The Combination of Synoeca-MP Antimicrobial Peptide with IDR-1018 Stimulates Proliferation, Migration, and the Expression of Pro-Regenerative Genes in Both Human Skin Cell Cultures and 3D Skin Equivalents
Source: Biomolecules. 2023 May 9;13(5):804. doi: 10.3390/biom13050804 (PMC10216143; doi:10.3390/biom13050804)
Supplement: Supplementary file 1 [file biomolecules-13-00804-s001.zip › biomolecules-2291598-supplementary.pdf]

biomolecules MDPI

Article

The Combination of Synoeca-MP Antimicrobial Peptide with IDR-1018 Stimulates Proliferation, Migration, and the Expression of Pro-Regenerative Genes in Both Human Skin Cell Cultures and 3D Skin Equivalents

Thuany Alencar-Silva [1], Rubén D. Díaz-Martín [1], Alessandra Zonari [2], Daniel Foyt [2], Mylieneth Guiang [2], Robert Pogue [1], Felipe Saldanha-Araujo [3,5], Simoni Campos Dias [1,6], Octavio Luiz Franco [1,4,5,7] and Juliana Lott Carvalho [1,8,*]

1 Programa de Pós-Graduação em Ciências Genômicas e Biotecnologia, Universidade Católica de Brasília, Brasília 71966-900, DF, Brazil
2 OneSkin, Inc., San Francisco, CA 94107, USA
3 Laboratório de Hematologia e Células-Tronco, Departamento e Farmácia, Universidade de Brasília, Brasília 70910-900, DF, Brazil
4 Programa de Pós-Graduação em Patologia Molecular, Universidade de Brasília, Brasília 70910-900, DF, Brazil
5 Programa de Pós-Graduação em Biologia Animal, Universidade de Brasília, Brasília 70910-900, DF, Brazil
6 S-Inova Biotech, Pós-Graduação em Biotecnologia, Universidade Católica Dom Bosco, Campo Grande 79117-900, MS, Brazil
7 Centro de Análises Proteômicas e Bioquímicas, Programa de Pós-Graduação em Ciências Genômicas e Biotecnologia, Universidade Católica de Brasília, Brasília 71966-900, DF, Brazil
8 Laboratório Interdisciplinar de Biociências, Faculdade de Medicina, Campus Darcy Ribeiro, Universidade de Brasília, Brasília 70910-900, DF, Brazil
* Correspondence: juliana.lott@unb.br

Citation: Alencar-Silva, T.; Díaz-Martín, R.D.; Zonari, A.; Foyt, D.; Guiang, M.; Pogue, R.; Saldanha-Araujo, F.; Dias, S.C.; Franco, O.L.; Carvalho, J.L. The Combination of Synoeca-MP Antimicrobial Peptide with IDR-1018 Stimulates Proliferation, Migration, and the Expression of Pro-Regenerative Genes in Both Human Skin Cell Cultures and 3D Skin Equivalents. *Biomolecules* **2023**, *13*, 804. https://doi.org/10.3390/biom13050804

Academic Editor: Francesc Rabanal Anglada

Received: 3 March 2023
Revised: 14 March 2023
Accepted: 16 March 2023
Published: 9 May 2023

Copyright: © 2023 by the authors. Licensee MDPI, Basel, Switzerland. This article is an open access article distributed under the terms and conditions of the Creative Commons Attribution (CC BY) license (https://creativecommons.org/licenses/by/4.0/).

Table S1. Primers and probes.

GENE	Forward sequence (5'-3')	Reverse sequence (5'-3')
GAPDH	CCCTGGACATCGAGATCGC	TGTGCTCCTGCTTGGACTC
KI67	TAACACCATCAGCAGGGAAAG	CTGCACTGGAGTTCCCATAAA
BCL2	CAAAGCTGCAGGCTGTTTAAG	GTCTGTCTGTGTGTGTGATGT
CXCR4	CATCCTCATCCTGGCTTTCTT	CACACCCTTGCTTGATGATTTC
CXCR7	GTGGTGGTCTGGGTGAATATC	ATGTAGCAGTGCGTGTCATAG
VEGFa	CTGTCTAATGCCCTGGAGCC	ACACGTCTGCGGATCTTGTA
FGF2	CAAGGACCCCAAGCGGCTGT	AGCTTGATGTGAGGGTCGCTCTT
HAS2	CTCGCAACACGTAACGCAAT	CAGTGCTCTGAAGGCTGTGT
ELN	AAGGCTGCCAAGTACGGAGT	CAAACTGGGCGGCTTTGG
MMP1	GAGCTTCCTAGCTGGGATATTG	ACTGGCCTTTGTCTTCTTTCT
GAPDH	Hs02786624_g1	
TGFb1	Hs00998133_m1	
TGFb3	Hs01086000_m1	
EPN3	Hs00203391_m1	
BLIMP 1	Hs00153357_m1	
COL1A1	Hs00164004_m1	
VEGF	Hs00900055_m1	
IL8	Hs00174103_m1	
